# Genome-Wide Identification and Expression Analysis of the Heavy-Metal ATPase (HMA) Gene Family Reveal Their Correlation with Copper and Cadmium Stress Adaptation in *Stevia rebaudiana*

**DOI:** 10.3390/cells15181646

**Published:** 2026-09-11

**Authors:** Dongbei Xu, Honglan Yu, Jingtian Xu, Yang Zhao, Jinbei Teng, Guilin Zhang, Xiuru Liu, Huanhuan Luo, Liwen Liao, Mengyuan Ran, Yanhong Jiang, Renlang Liu, Pingyang Liao, Kai Hou, Wei Wu, Xia Wang

**Affiliations:** 1 College of Agronomy, Sichuan Agricultural University, Chengdu 611130, China; pianyaoshuyan@163.com (H.Y.); rin2023@163.com (J.X.); 18710961851@163.com (Y.Z.); 13882223134@163.com (J.T.); 18784011757@163.com (G.Z.); wornas42@foxmail.com (X.L.); 17502816782@163.com (H.L.); 15196517084@163.com (L.L.); 19141368773@163.com (M.R.); 15823377056@163.com (Y.J.); liurenlang2023@163.com (R.L.); l.pingyang01@outlook.com (P.L.); hking@sicau.edu.cn (K.H.); ewuwei@sicau.edu.cn (W.W.); 2 Sichuan Provincial Forestry and Grassland Key Laboratory of Biodiversity Conservation and Sustainable Community Development in Giant Panda National Park, College of Chemistry and Life Science, Chengdu Normal University, Chengdu 611130, China

**Keywords:** *Stevia rebaudiana*, *HMA* gene, genome-wide identification, Cadmium stress, Copper stress

## Abstract

**Highlights:**

**What are the main findings?**

- Genome-wide identification revealed twelve *S. rebaudiana* SrHMA genes encoding stable, membrane-localized proteins with conserved E1-E2_ATPase, hydrolase and N-terminal HMA domains. These genes were phylogenetically classified into Zn/Co/Cd/Pb and Cu/Ag P1B-ATPase subfamilies, and are unevenly distributed across five chromosomes. Two segmental duplication events and a dicot-specific genome duplication event contributed to the expansion of this gene family. Expression profiling demonstrated tissue-specific regulation under copper and cadmium stress: cadmium upregulated most *SrHMA* genes in roots but suppressed them in leaves, whereas copper upregulated all genes in leaves and half of the genes in roots.

**What are the implications of the main findings?**

- This study provides the first systematic framework for the *SrHMA* gene family in *S. rebaudiana*, revealing its involvement in heavy-metal stress responses and offering candidate genes for breeding metal-tolerant varieties or for genetic improvement to reduce metal accumulation.

**Abstract:**

Heavy-metal ATPase (HMA) is widely involved in the absorption and transport of metal elements, including copper, zinc, and cadmium in plants. Although the characterization of HMA has been conducted in several plants, the research on the *HMA* gene in *Stevia rebaudiana* remains limited. In this study, twelve *SrHMA* genes were identified, encoding stable proteins mostly localized to the plasma membrane and possessing conserved E1-E2_ATPase and hydrolase domains, with one to three N-terminal HMA domains. Phylogenetic analysis classified them into two subfamilies (Zn/Co/Cd/Pb and Cu/Ag P1B-ATPases). The genes are unevenly distributed across five chromosomes, with two segmental duplication events and an independent genome duplication event specific to dicots, and their promoters harbor abundant stress-responsive elements. Expression profiling under copper and cadmium stresses showed distinct tissue specificity: cadmium stress upregulated most genes in roots but suppressed them in leaves, whereas copper stress upregulated all genes in leaves and half of the genes in roots. This study represents the first systematic analysis of the *SrHMA* gene family, revealing its potential involvement in heavy-metal stress responses and providing candidate genes for future functional characterization and breeding of *S. rebaudiana* varieties with enhanced metal tolerance.

## 1. Introduction

In recent years, the rapid industrialization, intensive agriculture and urban sprawl have aggravated soil heavy-metal contamination [1], posing an increasing threat to the quality and safety of economic crops. More seriously, plant-derived heavy metals undergo trophic transfer and bioaccumulation along the food chains, thus gravely endangering both ecological safety and public health [2,3,4].

Notably, trace metal ions, including essential metals such as copper (Cu), zinc (Zn), and manganese (Mn) as well as nonessential heavy metals like cadmium (Cd) and lead (Pb), can be absorbed by plants [5,6,7]. Essential metals are required for normal plant physiological and biochemical processes. However, both the over-accumulation of essential metals and the presence of nonessential heavy metals can cause toxic injuries to plants. Among these, Cu and Cd are of particular concern, as Cu is phytotoxic at elevated concentrations and Cd is highly toxic even at low levels, and both are widely distributed in the environment [8,9,10]. Excess Cu^2+^ and Cd^2+^ act as high-level stress factors that trigger systemic physiological imbalances through common mechanisms, including ROS bursts and disruption of ion homeostasis. At the cellular level, root meristematic cells suffer direct growth inhibition, whereas mesophyll cells undergo photosynthetic collapse. At the organellar level, chloroplasts and mitochondria are preferentially targeted by oxidative damage, due to their high electron transport activity, leading to structural degradation [11]. Therefore, exploring the heavy-metal stress tolerance mechanism in plants is of great significance to improve their adaptability to adverse environments.

To counteract the adverse effects of heavy-metal toxicity, plants have evolved diverse regulatory strategies to prevent excessive heavy-metal accumulation in plant tissues, including but not limited to restricted uptake, cellular extrusion, compartmentalization, chelation and detoxification [6,12]. Most of the above processes rely on a variety of transmembrane transporters to dynamically maintain the homeostasis of different metal ions in planta. Accordingly, a large number of metal-transport-related proteins have been extensively identified to date, including heavy-metal ATPases (HMA), copper transporters (COPT), yellow stripe-like proteins (YSL), cation diffusion facilitator proteins (CDF), Zn-regulated transporter-like proteins (ZNT/ZIP), metal tolerance proteins (MTP), ATP-binding cassette proteins (ABC), and natural resistance-associated macrophage proteins (NRAMP), which have been shown to constitute an intricate transport network that coordinates the redistribution of various heavy metals and maintains heavy-metal homeostasis within plant cells [13,14].

HMA, also known as P1B-ATPase, is a member of the large P-type ATPase family and plays a crucial role in regulating the absorption and transport of diverse heavy-metal ions including Cu^2+^ and Cd^2+^ across membranes by using the energy resulting from ATP hydrolysis [15,16,17]. HMA proteins typically contain 6 to 8 transmembrane helices, a histidine proline locus, and a CPx/SPC motif containing three functional domains, namely P, N, and A, which are respectively responsible for enzyme phosphorylation, nucleotide binding and energy transduction, as well as N- and C-terminal soluble metal-binding domains (metal-binding domains, MBD) [15,18]. According to the types of substrate metal ions transported by different HMAs, HMAs can be classified into two subcategories, namely the Zn/Co/Cd/Pb P1B-ATPase subcategory and the Cu/Ag P1B-ATPase subcategory [15]. Based on the substrate specificity and conserved amino acid sequences in the transmembrane segments, P1B-ATPases are further classified into seven subgroups, namely P1B-1 to P1B-7 [15]. However, in plants, only three subgroups, P1B-1, P1B-2, and P1B-4, are present [19]. *HMA* genes are widely present in plants, among which the HMA members in *Arabidopsis thaliana* [20], *Hordeum vulgare* [21], *Zea mays* [22], *Oryza sativa* [22], *Glycine max* [23], *Populus tomentosa* [24], and *Hydrangea macrophylla* [25] have been systematically identified at the genomic level. Studies have shown that HMA members can promote plant growth and development by maintaining the homeostasis of ions, and can also enhance tolerance to and accumulation of heavy metals. For instance, AtHMA3 is located in the vacuole membrane, mediating the sequestration of Cd, Zn, and Pb, etc., into the vacuole, thereby enhancing the plant’s tolerance to heavy metals [26]. The *IlHMA2*-silenced line is sensitive to high Cd levels, with severe growth retardation and significantly reduced Cd and Zn concentrations in the xylem, indicating that *IlHMA2* plays a key role in the root-to-shoot transport of Zn and Cd and in maintaining Zn homeostasis, thereby enhancing its tolerance to Cd [27]. The *HMA* gene also plays a core role in copper transport and detoxification: *AtHMA1*, *AtHMA6*, and *AtHMA8* are all involved in chloroplast copper homeostasis [28,29]; under copper stress, different *PtHMA* genes (*PtHMA5*-*PtHMA8*) show differential expression patterns at the organ level (e.g., roots and leaves) and presumably in specific cell types within these organs, jointly responding to copper toxicity [24]. The expression of *AtHMA5* is specifically induced by copper and is mainly expressed in roots, playing a significant role in copper detoxification of the root system [17]. These findings highlight the crucial role of the *HMA* gene family in plant tolerance to heavy-metal stress and its potential application in crop resistance breeding.

*Stevia rebaudiana* Bertoni is a perennial herbaceous plant belonging to the Asteraceae family. Its active ingredient, stevioside, serves as a natural, safe, low-calorie, and high-potency sweetener. It has been widely applied in the global food and pharmaceutical industries and has been certified by international regulatory bodies such as the European Food Safety Authority (EFSA) and the Codex Alimentarius [30,31]. With the growing consumer preference for healthy diets, its market demand continues to grow. It is estimated that the global market value will reach approximately USD 818 million by 2024 [32]. However, during cultivation, *S. rebaudiana* plants are frequently exposed to various abiotic stresses. Among them, heavy-metal pollution has become a key limiting factor affecting the growth and metabolite accumulation of *S. rebaudiana*. It is worth noting that the main production areas of *S. rebaudiana* are located in arid and semi-arid regions, where precipitation is low and evaporation is high. Heavy-metal elements are not readily leached or transported and tend to accumulate in the soil, resulting in relatively high background levels of heavy metals [33,34]. Copper (Cu) and cadmium (Cd), as major pollutants, have been shown to significantly affect the physiological metabolism and growth of *S. rebaudiana* [35,36]. Existing studies have shown that plant P1B-ATPase plays a central role in regulating metal ion transport and detoxification [18]. Investigating the expression and function of *HMA* genes in *S. rebaudiana* is therefore essential for ensuring raw material safety and enabling sustainable production.

Despite extensive studies on the functions of the *HMA* gene family in model plants and crops, the genome-wide identification of HMA members in the important cash crop *S. rebaudiana*, as well as their expression regulation and specific mechanisms underlying responses to copper and cadmium stress, remain unclear. Therefore, this study aimed to systematically identify the members of the *S. rebaudiana HMA* gene family at the whole-genome level, and to analyze their phylogenetic relationships, conserved domains, and expression characteristics, with a particular focus on screening key candidate genes responsive to copper and cadmium stress. These analyses of the *SrHMA* genes are expected to facilitate future genetic engineering strategies, including regulating their expression to enhance tolerance or blocking uptake pathways to reduce the accumulation of heavy metals such as copper and cadmium in *S. rebaudiana*. Ultimately, this study provides a molecular and theoretical foundation for breeding new *S. rebaudiana* varieties with enhanced resistance to heavy-metal pollution.

## 2. Materials and Methods

### 2.1. Genome-Wide Identification and Characterization of Srhma Genes

The corresponding HMA protein sequences were obtained from the *A. thaliana* TAIR database (https://www.arabidopsis.org/; accessed on 27 March 2026) and the rice database (http://rice.uga.edu/; accessed on 27 March 2026), respectively. Subsequently, using the above-mentioned protein sequences as queries, the BLASTP algorithm in TBtools (version 2.467) was employed for homologous sequence alignment to preliminarily screen candidate genes. The characteristic domains of the HMA protein family including E1-E2_ATPase (PF00122), Hydrolase (PF00702), and HMA (PF00403) were downloaded from the Pfam database (http://pfam-legacy.xfam.org/; accessed on 28 March 2026) to identify SrHMA proteins via the Simple HMM Search tool in TBtools. Only hits with an E-value < 1 × 10^−5^ were retained from each domain search, and the intersection of the three domain-based results was considered as the candidate SrHMA proteins. The results from the two approaches were merged, and duplicate sequences were removed. The domain structures were further verified using the CDD (http://www.ncbi.nlm.nih.gov/cdd/; accessed on 28 March 2026) and Pfam (http://pfam.xfam.org/search/sequence; accessed on 28 March 2026) databases. After removing sequences without conserved domains, incomplete structures, and redundant sequences, candidate genes were obtained. The basic physicochemical parameters of the candidate SrHMA proteins were determined using the ExPASy online platform (https://web.expasy.org/protparam/; accessed on 29 March 2026). Subcellular localization was predicted using the WoLF PSORT website (https://wolfpsort.hgc.jp/; accessed on 30 March 2026).

### 2.2. Analysis of Homologous Amino Acid Sequences of the SrHMA Genes and Prediction of Protein Structure

Multiple-sequence alignment of protein sequences of HMA members from *A. thaliana*, *O. sativa*, and *S. rebaudiana* was performed using the MUSCLE algorithm implemented in MEGA11. Subsequently, a phylogenetic tree was constructed using the Neighbor-Joining (NJ) method with 1000 bootstrap replicates in MEGA11, and the tree was visualized using Evolview (https://www.evolgenius.info/evolview-v2/; accessed on 19 August 2026). Protein secondary structures were predicted using SOPMA (https://npsa-prabi.ibcp.fr/cgi-bin/npsa_automat.pl?page=npsa_sopma.html; accessed on 21 August 2026), and tertiary structures were predicted using SWISS-MODEL (https://swissmodel.expasy.org/interactive; accessed on 3 April 2026) with default parameters.

### 2.3. Gene Structure, Domain and Conserved Motif Analysis

The introns and exons of each *SrHMA* gene were identified using TBtools. The conserved motifs of *S. rebaudiana* HMA proteins were analyzed using the MEME website (https://meme-suite.org/meme/tools/meme; accessed on 18 August 2026) with default parameters, and the maximum number of motifs was set to 20. The conserved domains were analyzed using Batch CD-Search with default parameters. All results were integrated and visualized using TBtools.

### 2.4. Chromosome Position and Collinearity Analysis

The chromosomal distribution of *SrHMA* genes was obtained from the GFF3 file of the *S. rebaudiana* genome and visualized using TBtools. Gene duplication events and collinearity were analyzed using MCScanX with default parameters. To explore the collinearity between orthologous *HMA* genes in *S. rebaudiana* and those in dicot plants (*A. thaliana*, *Helianthus annuus*, and *G. max*), as well as in monocot plants (*Z. mays* and *O. sativa*), a collinearity plot was constructed using MCScanX and visualized with TBtools. The Ka/Ks ratio between non-synonymous substitution (Ka), synonymous substitution (Ks), and collinear gene pairs (Ka/Ks) was calculated by TBtools.

### 2.5. Analysis of Cis-Regulatory Elements of SrHMA Genes

The 2000 bp promoter sequences upstream of the start codons of the *SrHMA* genes were extracted using TBtools. Cis-regulatory elements were identified using PlantCARE (https://bioinformatics.psb.ugent.be/webtools/plantcare/html/; accessed on 18 August 2026), and the results were visualized using TBtools.

### 2.6. Prediction of SrHMA Protein–Protein Interactions and Analysis of miRNA Regulatory Networks

A protein–protein interaction (PPI) network of SrHMA proteins was constructed using the STRING database (https://string-db.org/cgi/input.pl; accessed on 19 August 2026) to identify potential interacting partners, which may help generate hypotheses about the functional associations and regulatory networks of SrHMA proteins.

To elucidate the post-transcriptional regulatory network of the *SrHMA* gene family, miRNA targets were predicted using the psRNATarget online tool (https://www.zhaolab.org/psRNATarget/; accessed on 20 August 2026). Furthermore, given the limited miRNA information available for *S. rebaudiana*, *A. thaliana*, which possesses the most comprehensive miRNA annotations among model plants, was used as a reference for homology-based identification of potential miRNAs targeting *SrHMA* genes. The resulting regulatory network was visualized using Cytoscape (version 3.9.1) [37].

### 2.7. RNA-Seq Data Analysis of the SrHMA Genes in S. rebaudiana

RNA-seq data, including different developmental stages of leaves and tissues of *S. rabaudiana*, were downloaded from the SRA database at NCBI (https://www.ncbi.nlm.nih.gov/; BioProject ID: PRJNA705537; accessed on 7 April 2026). The tissues included: root at the seedling stage (RS), stem at the seedling stage (SS), leaf at the seedling stage (LS), leaf at the vegetative stage (LV), leaf at the bud stage (LB), leaf at the initial flowering stage (LIF), and leaf at the peak flowering stage (LPF). The raw transcriptome data were processed using a log_2_(TPM + 1) transformation, and expression levels were visualized as a heatmap using the HeatMap tool in TBtools.

### 2.8. Materials and Treatments

The experiment used the *S. rebaudiana* variety ‘Puxing No. 6’. Shoot tip cuttings from healthy *S. rebaudiana* plants were excised and planted in a standard potting mix. The cuttings were cultivated in a growth chamber under a photoperiod of 16 h light/8 h dark at 23–25 °C. After two weeks of cultivation, healthy seedlings of uniform size were selected. The roots were cleaned with sterile water to remove soil, and the seedlings were then transferred to 1/2 Hoagland nutrient solution for a two-day recovery period. The roots of the plants were respectively immersed in 100 μM CdCl_2_ [38] and 721 μM CuSO_4_ solutions for treatment. Samples, including the second pair of leaves from the top and root tissue samples at six time points: 0, 1, 3, 6, 12, and 24 h after treatment, were collected, immediately frozen in liquid nitrogen, and stored at −80 °C for further use. Three biological replicates were set up for each treatment.

### 2.9. RNA Extraction and Quantitative Real-Time PCR

Total RNA was extracted using the RNAiso Plus kit (cat. no. 9109, Takara Bio Inc., Otsu, Shiga, Japan) following the manufacturer’s instructions. The concentration and purity of the RNA samples were measured with a NanoDrop 2000 spectrophotometer (Thermo Fisher Scientific, Waltham, MA, USA), while RNA integrity was assessed by agarose gel electrophoresis. cDNA was synthesized using the HiScript^@^ II 1st Strand cDNA Synthesis kit (cat. no. R212, Vazyme Biotech Co. Ltd., Nanjing, China) according to the provided protocol. Gene-specific primers for the *SrHMA* genes were designed with Primer 5.0 software (Supplemental Table S1), with the *β-actin* gene (AF548026) serving as an internal reference. RT-qPCR was performed using the NovoStart^®^ Fast SYBR qPCR SuperMix kit (cat. no. E301-01A, Novoprotein Scientific Inc., Suzhou, China). The expression levels of each gene at different treatment time points were calculated using the 2^−∆∆Ct^ method, and statistical significance of differences was analyzed with SPSS software.

## 3. Results

### 3.1. Identification and Characterization of the SrHMA Gene Family in S. rebaudiana

To identify members of the *HMA* gene family in *S. rebaudiana*, the protein sequences of eight *A. thaliana* and nine *O. sativa* HMAs (Supplemental Table S2) were first used as queries to perform BLASTP searches against the *S. rebaudiana* protein database, yielding initial candidate sequences. Additionally, the HMM search was performed using the conserved domain of HMA proteins to further identify the SrHMA proteins, and then all non-redundant SrHMA proteins from the above-mentioned approaches were merged and these candidates were further examined for conserved domains (E1-E2_ATPase, Hydrolase, and HMA) using the Pfam (http://pfam.xfam.org/search/sequence) and CDD (http://www.ncbi.nlm.nih.gov/cdd/) databases. After removing sequences lacking typical domains and those with incomplete structures, 13 putative genes were initially identified and designated SrHMA1 to SrHMA13. However, upon further verification, *SrHMA13* was excluded from subsequent analyses due to its incomplete coding sequence caused by chromosome assembly gaps. Thereafter, 12 non-redundant *SrHMA* genes were ultimately retained (Table 1). Analysis of the physicochemical properties of the *SrHMA* family members revealed that the proteins range from 763 to 1149 amino acids in length, with SrHMA12 being the largest and SrHMA3 the smallest. The molecular weights (MWs) varied between 82,105.74 and 127,330.27 Da, following the same trend. The predicted isoelectric points (pI) ranged from 5.23 to 7.53. Eleven proteins were acidic (pI < 7), while one was basic (pI > 7), indicating that most SrHMA proteins are acidic. The instability index (II) values ranged from 28.28 (SrHMA6) to 42.19 (SrHMA3). With the exception of SrHMA3, which exhibited an instability index of 42.19 exceeding the threshold of 40.0 for unstable proteins, all other SrHMA members had instability indices below 40.0, indicating they are predicted to be stable. The grand average of hydropathicity (GRAVY) values were negative only for SrHMA6 and SrHMA12, suggesting hydrophilic character, whereas the remaining proteins were hydrophobic. Subcellular localization predictions showed that most proteins are localized to the plasma membrane, except SrHMA3 (chloroplast) and SrHMA9 (vacuole membrane).

### 3.2. Phylogenetic Analysis of the SrHMA Genes in S. rebaudiana

**Figure 1:**
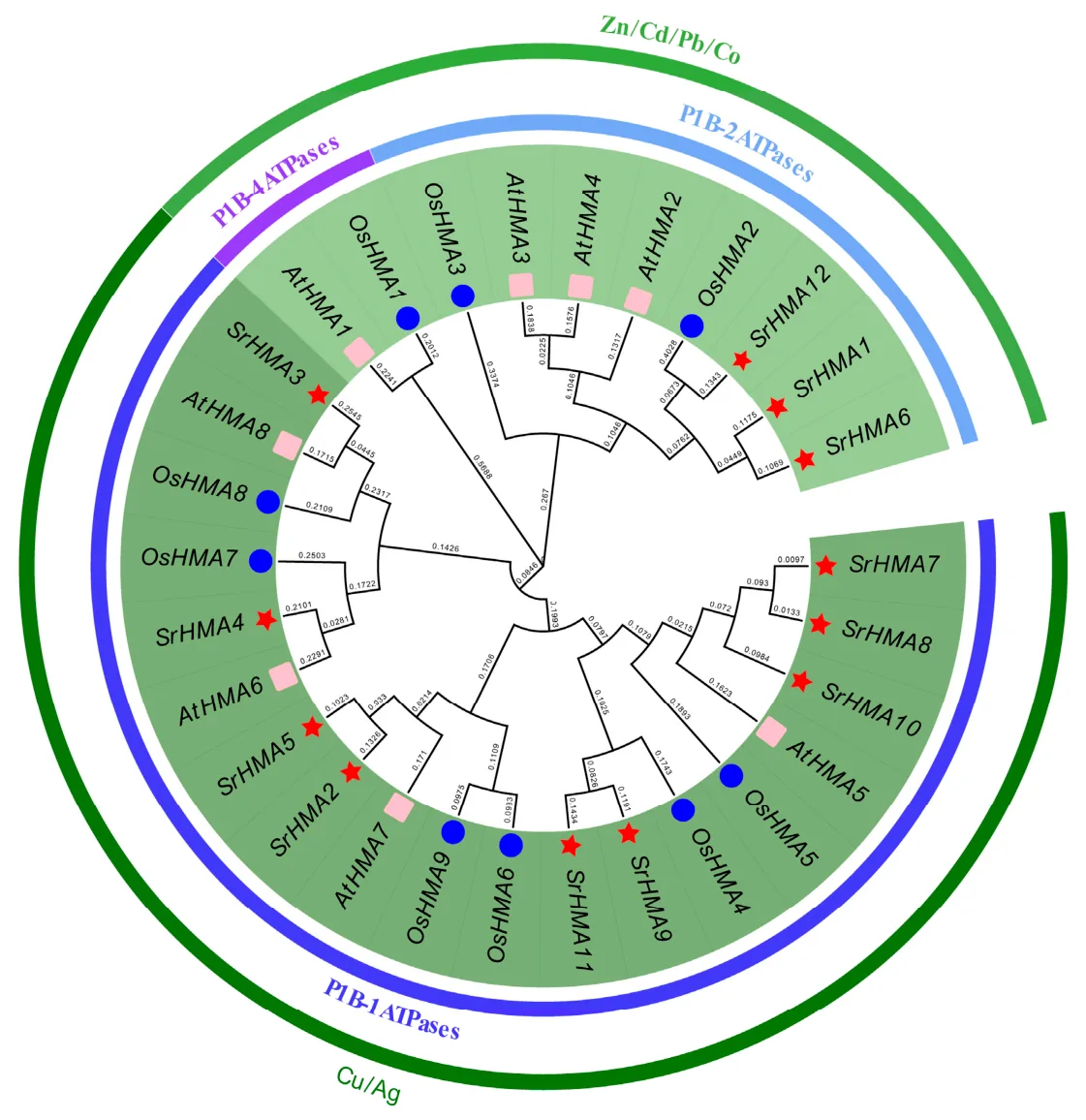
Phylogenetic analysis of HMA proteins from *S. rebaudiana*, *A. thaliana*, and *O. sativa*. The full-length amino acid sequences of AtHMA, OsHMA and SrHMA proteins were compared using MEGA11, and the phylogenetic tree was constructed with the neighbor-joining (NJ) method. The phylogenetic tree is subdivided into two subgroups, highlighted in distinct colors. Red stars, pink squares, and blue circles indicate HMA proteins from *S. rebaudiana*, *A. thaliana*, and *O. sativa*, respectively. (Sr: *S. rebaudiana*; At: *A. thaliana*; Os: *O. sativa*).

To clarify the phylogenetic relationships of the *HMA* genes in *S. rebaudiana*, a phylogenetic tree was constructed using HMA protein sequences from *A. thaliana* and *O. sativa*. The resulting phylogenetic tree (Figure 1) shows that the twelve *SrHMA* genes can be divided into two major clades. Three *SrHMA* genes (*SrHMA1*, *SrHMA6*, and *SrHMA12*) belong to the Zn/Co/Cd/Pb P_1_B-ATPase subgroup, while the remaining nine fall into the Cu/Ag P_1_B-ATPase subgroup. This classification is consistent with that of *A. thaliana* and *O. sativa HMAs*, indicating that the *HMA* family emerged prior to the divergence of dicot and monocot plants.

### 3.3. Analysis of Homologous Amino Acid Sequences of the SrHMA Gene and Prediction of Protein Structure

The HMA protein sequences in *S. rebaudiana*, *A. thaliana* and *O. sativa* were compared, and the results showed that in *S. rebaudiana*, SrHMA1, SrHMA6, and SrHMA12 belonged to the P1B-2 ATPase subgroup, while the remaining SrHMA members belonged to the P1B-1 ATPase subgroup (Figure 1). No SrHMA protein members were classified into the P1B-4 ATPase subgroup. Except for SrHMA3, which lacks the TM5 (YN[X]_4_P) motif, all members of the P1B-1 ATPase subgroup contain the characteristic motifs TM4 (CPC), TM5 (YN[X]_4_P), and TM6 (M[XX]SS) ("X" can refer to any residue). Members of the P1B-2 ATPase subgroup all contain conserved lysine residues in the TM4 (CPC), TM5 (K), and TM6 (DXTG) motifs (Supplemental Figure S1).

The results of secondary structure prediction revealed that SrHMA proteins are predominantly composed of α-helices (36.64–45.35%) and random coils (30.73–47.00%), along with extended strands (11.66–17.33%) and a small proportion of β-turns (4.70–7.29%) (Supplemental Table S3 and Supplemental Figure S2). This structural architecture provides a molecular basis for the integration of structural stability and functional diversity, which may facilitate the efficient sequestration and detoxification of heavy-metal ions in the environment.

### 3.4. Gene Structure, Conserved Domain and Motif Analysis of SrHMA Members

**Figure 2:**
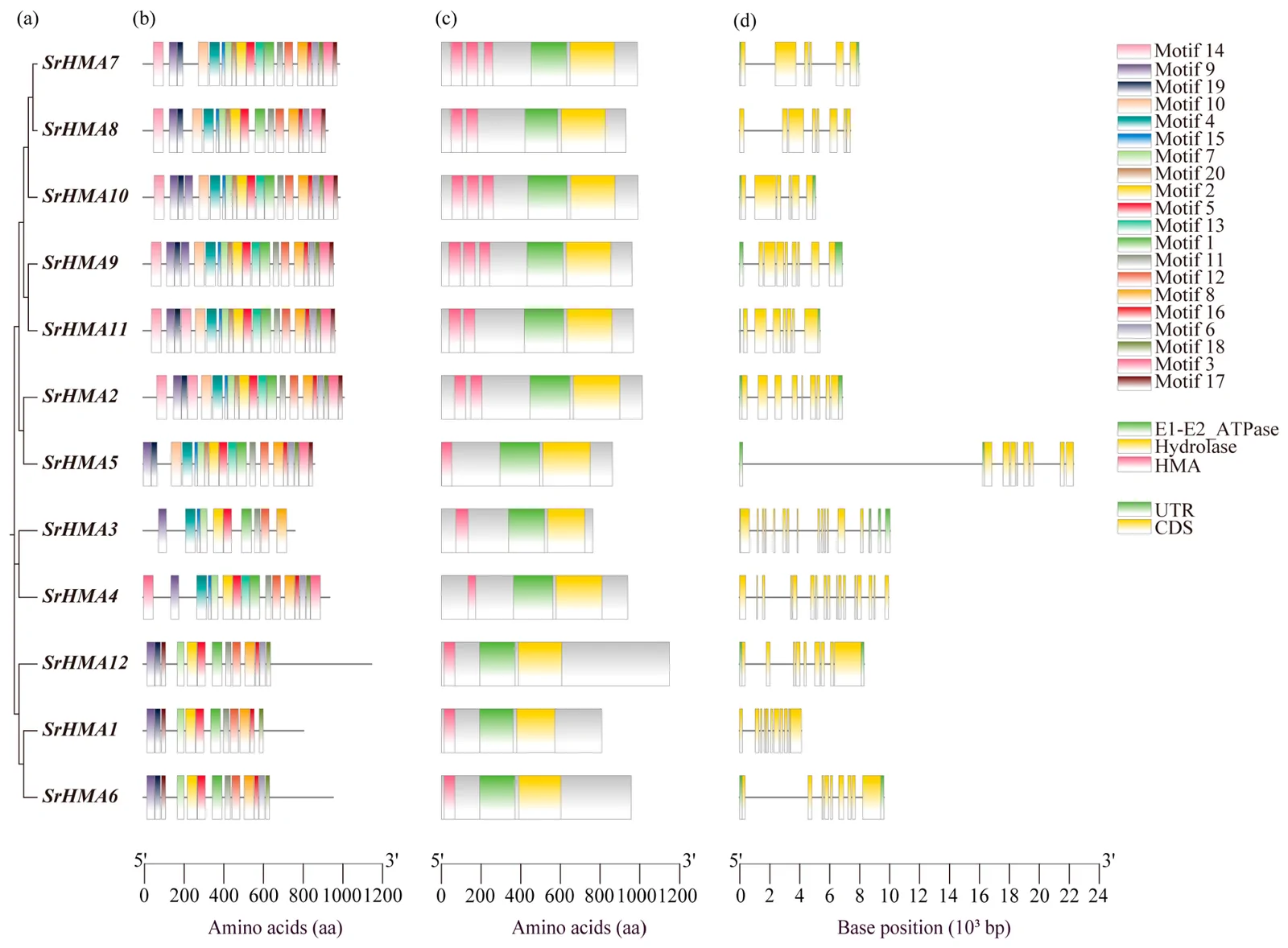
Phylogenetic clustering, conserved motifs, protein domains, and gene structure of the 12 *SrHMA* genes. (**a**) Phylogenetic relationships; (**b**) protein motifs; (**c**) protein domains; and (**d**) gene structure. The horizontal scale represents the length of the gene/amino acid sequence. UTR: untranslated region, CDS: coding sequences.

A phylogenetic tree was constructed using the full-length SrHMA protein sequences (Figure 2a). Based on the phylogenetic analysis, the twelve SrHMA proteins were classified into two groups: Zn/Co/Cd/Pb SrHMAs (SrHMA1, SrHMA6, and SrHMA12) and Cu/Ag SrHMAs (SrHMA2, SrHMA3, SrHMA4, SrHMA5, SrHMA7, SrHMA8, SrHMA9, SrHMA10, and SrHMA11). A total of 20 conserved motifs in SrHMA proteins were predicted using MEME (Figure 2b), showing that all SrHMAs contain Motif 1, Motif 2, Motif 4, Motif 7, Motif 8, Motif 9, Motif 10, and Motif 17. To further reveal the structural diversity and functional features of SrHMA proteins, conserved domains and gene structures of the *SrHMA* gene family were analyzed (Figure 2c,d). As shown, all *SrHMA* family members contain an E1-E2_ATPase domain, which provides energy for ion transport, a hydrolase domain involved in membrane hydrolysis, and 1–3 HMA domains at the N-terminus, with the number varying among members: SrHMA7/9/10 contain three, SrHMA2/8/11 contain two, and SrHMA1/3/4/5/6/12 contain only one. The number of exons in *SrHMA* genes ranges from 6 to 17, with *SrHMA7* and *SrHMA10* having the fewest (6 exons) and *SrHMA4* containing the most (17 exons). Notably, an unusually long intron was observed in the gene structure of *SrHMA5* (Figure 2d). Based on the currently available genome assembly of *S. rebaudiana*, this region indeed contains a long intronic sequence. However, given the highly repetitive nature of the *S. rebaudiana* genome, which may affect the accuracy of gene structure prediction, it remains unclear whether this long intron represents a genuine structural feature or an assembly artifact. Thus, a higher-quality reference genome, such as a telomere-to-telomere (T2T) assembly, would be required to definitively resolve the authenticity of this long intron in the future.

### 3.5. Chromosome Distribution, Homology and Evolutionary Analysis of SrHMAs

**Figure 3:**
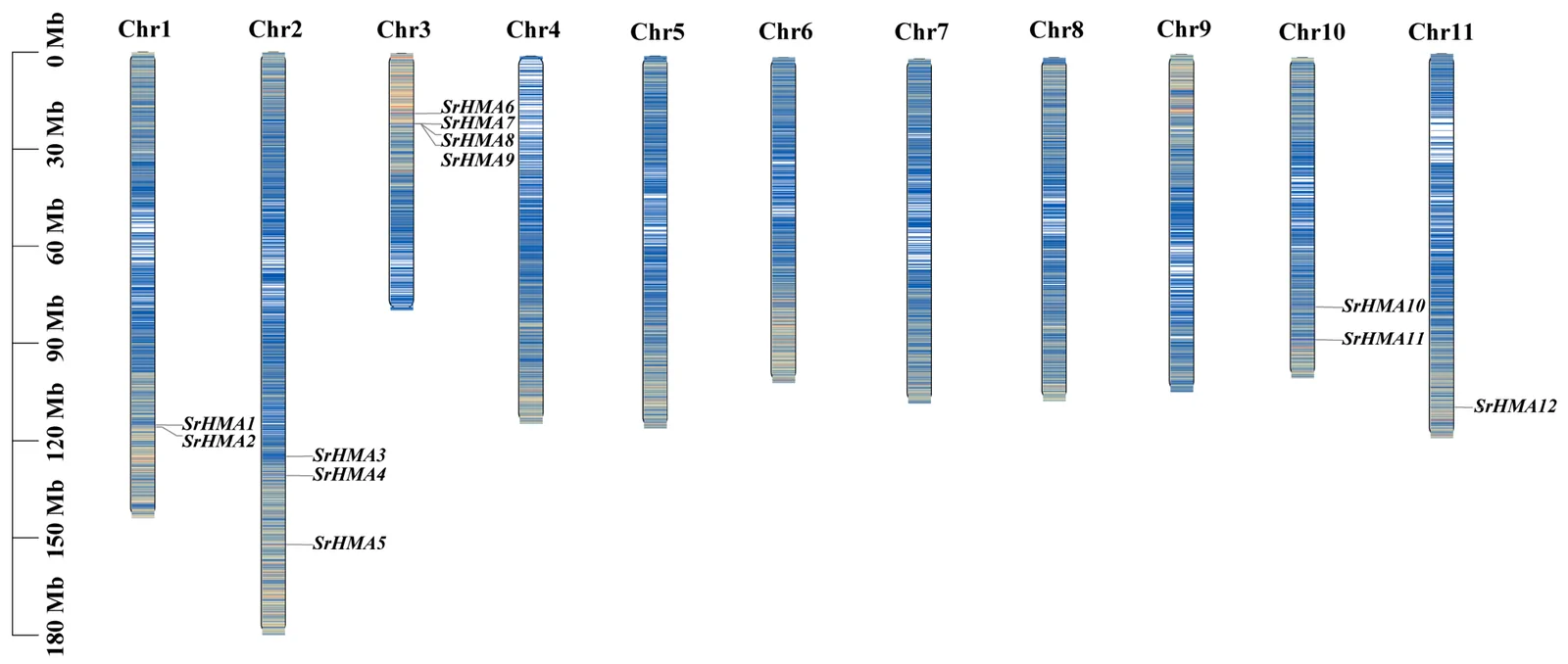
Distribution of the twelve *SrHMA* genes across the 11 chromosomes of *S. rebaudiana*. Note that *SrHMA* genes are located on only five chromosomes. The vertical axis represents chromosome length (Mb), and chr1–chr11 denote the 11 chromosomes of *S. rebaudiana*. The *SrHMA* genes are marked in black.

Chromosomal localization analysis revealed that the twelve identified *SrHMA* family members are distributed across five chromosomes (Figure 3). Chromosome 3 (Chr3) contains the highest number of members, with four *SrHMA* genes (*SrHMA6*, *SrHMA7*, *SrHMA8*, and *SrHMA9*), while Chr11 carries only one member, *SrHMA12*.

**Figure 4:**
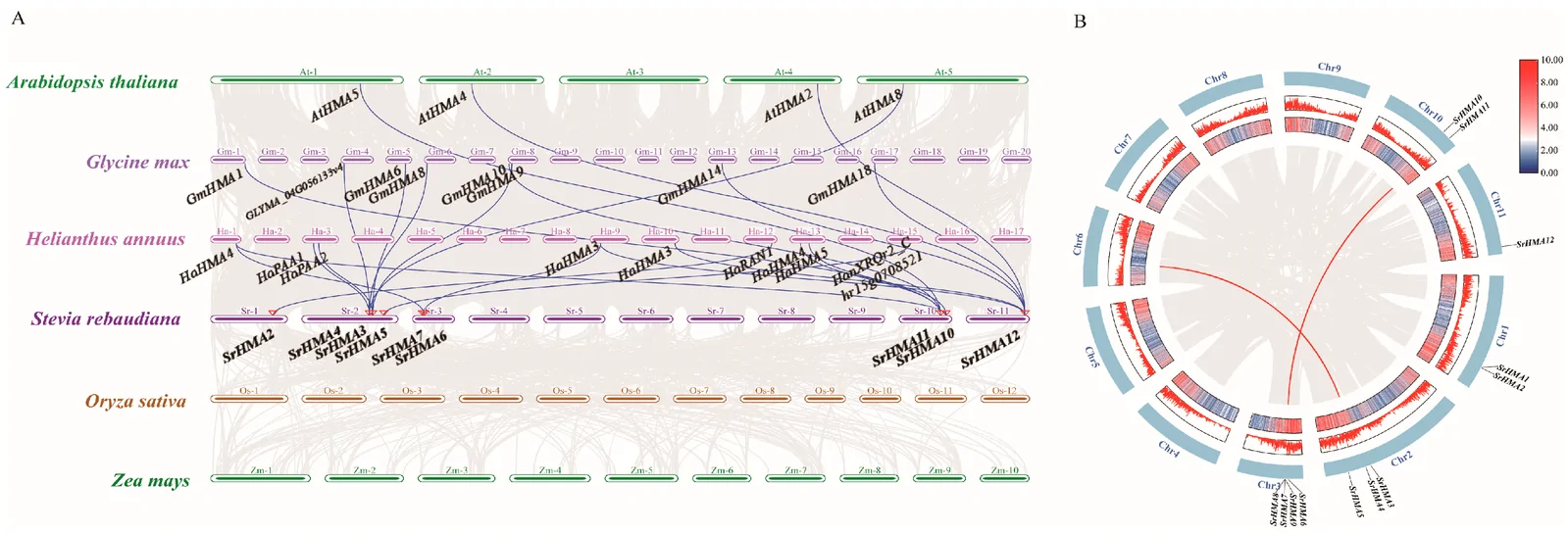
Collinearity analysis of the *SrHMA* gene family. (**A**) Syntenic relationships of HMA members between *S. rebaudiana* and five representative plant species. The gray lines indicate gene duplication events that occurred across all genes between the *S. rebaudiana* genome and the genomes of the five species during evolution. The blue lines highlight gene duplications specifically between *HMA* genes in *S. rebaudiana* and *HMA* genes in the five species. Red open triangles indicate *SrHMA* genes from *S. rebaudiana*. The species names are shown in different colors to distinguish the plant species. (**B**) Intra-genomic synteny analysis of *SrHMA* genes in *S. rebaudiana*. The innermost circle (Chr1–Chr11) represents the 11 chromosomes of *S. rebaudiana*, while the outermost circle shows the distribution of the twelve *SrHMA* genes across the chromosomes. The gray lines indicate genome-wide gene duplication events among all genes in the *S. rebaudiana* genome, and the red lines highlight segmental duplication events specific to the *SrHMA* genes, including *SrHMA11*–*SrHMA7* and *SrHMA4*–*Streb.6G009190.1*.

Synteny analysis between *S. rebaudiana* and the genomes of *H. annuus*, *A. thaliana*, *G. max*, *Z. mays*, and *O. sativa* revealed four syntenic gene pairs with *A. thaliana*, eight with *G. max*, and the highest number (11) with the closely related *H. annuus* (Figure 4A). No synteny was detected between *S. rebaudiana* and monocots such as *O. sativa* or *Z. mays*. These results suggest that the *HMA* gene family underwent independent and extensive genomic duplication events in dicots after the divergence of dicotyledonous and monocotyledonous plants, leading to genomic rearrangements and an increase in paralogous gene numbers in dicots, while the family remained relatively conserved in monocots.

Genome duplication is a key mechanism driving the expansion of genetic material. Based on their organizational patterns in the genome, duplicated genes can be classified into the following five phylogenetic types: singleton duplicates, dispersed duplicates, tandem duplicates, proximal duplicates, and segmental duplicates [39]. Based on the intraspecific collinearity analysis of *S. rebaudiana*, we identified two segmental duplication events in the *SrHMA* gene (*SrHMA11*-*SrHMA7, SrHMA4*-*Streb.6G009190.1*). The Ka/Ks values of the two duplicated gene pairs were both less than 1, indicating that *SrHMA* genes were generally under purifying selection (Figure 4B, Supplemental Table S4). A similar trend has been observed in other species such as *H. macrophylla* [25], *Fagopyrum tataricum* [40], and *Triticum aestivum* [41].

### 3.6. Analysis of Cis-Regulatory Elements in the SrHMA Genes of S. rebaudiana

**Figure 5:**
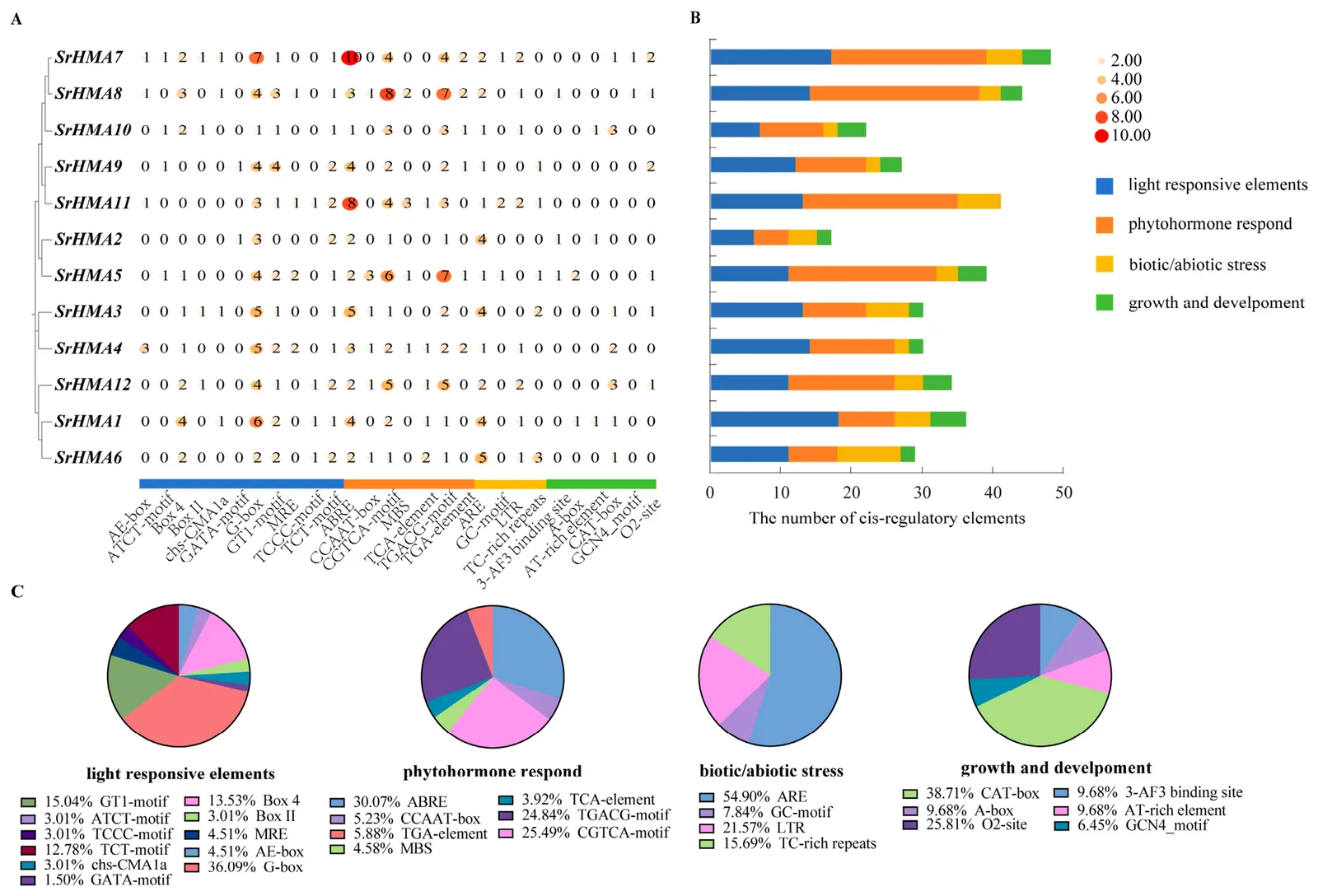
Analysis of cis-regulatory elements of the *SrHMA* gene family members in *S. rebaudiana*. (**A**) The number of different cis-regulatory elements in the *SrHMA* genes. (**B**) The number of cis-regulatory elements in each category. (**C**) The ratio of different cis-regulatory elements in each category.

Gene expression is directly regulated by cis-acting elements within promoter regions. To investigate the functional characteristics of the *SrHMA* gene family in *S. rebaudiana*, we analyzed the promoter sequences of each member for cis-acting elements (Figure 5A–C). The results revealed a variety of functional cis-regulatory elements in the promoter regions of *SrHMA* genes, which were classified into four major categories: light-responsive, hormone-responsive, stress-related, and plant growth-related elements. Light-responsive elements such as Box 4, TCT-motif, GT1-motif, MRE, and G-box were detected in the promoters of all examined genes and occurred at significantly higher frequencies than other types of elements. This strongly suggests widespread involvement of this gene family in light signaling pathways. Furthermore, the *SrHMA* genes were found to contain abundant abiotic stress-responsive elements. All promoters harbored multiple regulatory elements associated with environmental stress and hormone responses, including anaerobic stress-responsive elements (ARE, GC-motif), drought-inducible elements (MBS), low-temperature response elements (LTR), auxin response elements (TGA-box, TGA-element), methyl jasmonate-responsive motifs (CGTCA-motif, TGACG-motif), gibberellin response elements (P-box, TATC-box), and abscisic acid response elements (ABRE). Notably, regulatory elements related to growth and development such as meristem-specific activation elements (CAT-box, O2-site) were detected at significantly lower frequencies, and were even absent in SrHMA11. These findings indicate that *SrHMA* genes are predominantly involved in stress response processes, while also potentially playing roles in plant development.

### 3.7. Prediction of SrHMA Protein–Protein Interactions and Analysis of miRNA Regulatory Networks

**Figure 6:**
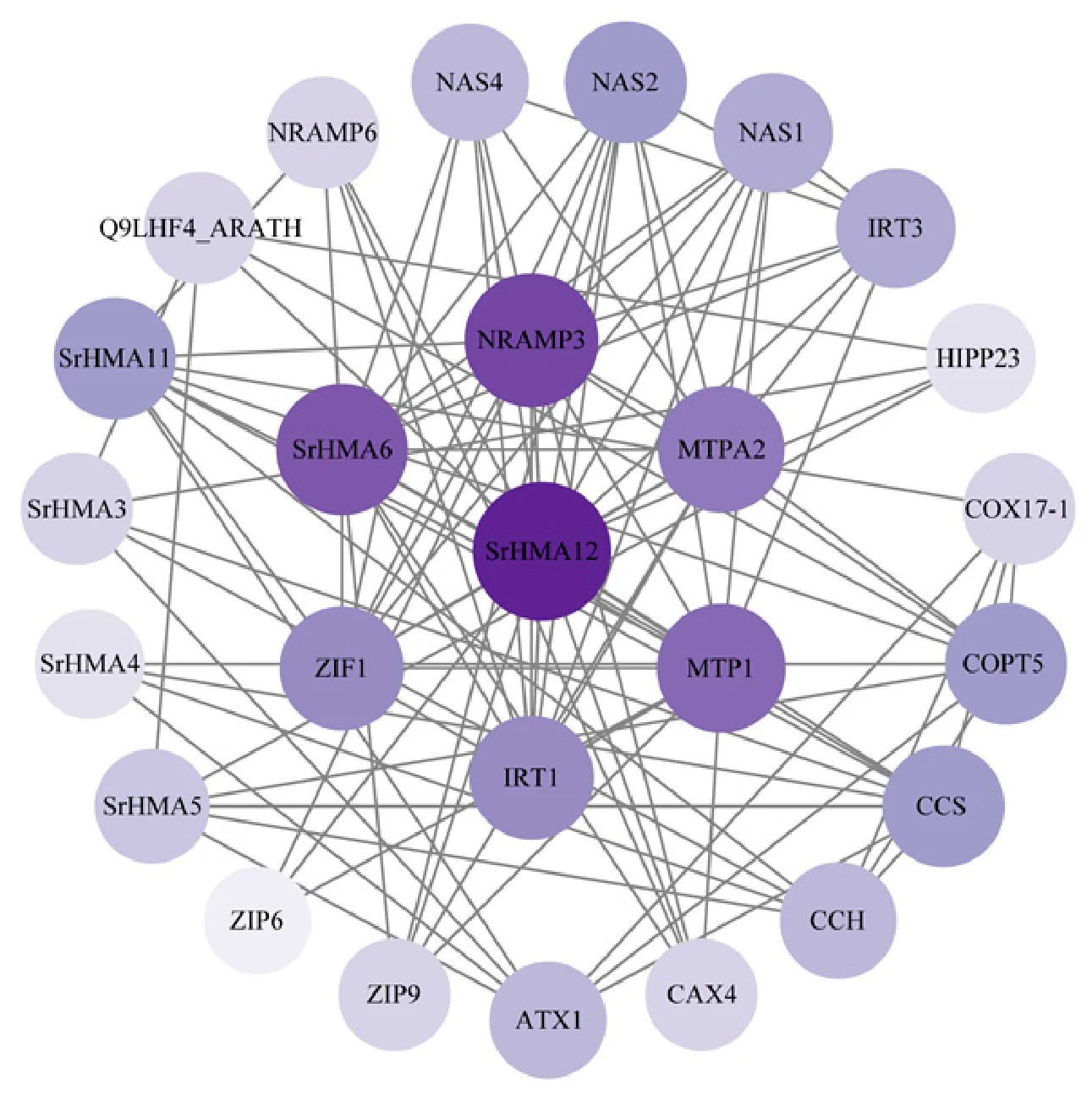
Prediction of proteins interacting with SrHMA proteins. The circles represent indicated proteins. The purple color intensity of each circle corresponds to the count of interacting proteins, where a darker shade indicates a greater number of interaction partners for the candidate protein, and a lighter shade indicates a smaller number of interaction partners. All circles are of equal size. Grey lines represent protein–protein interactions and do not have a quantitative scale.

PPI prediction revealed that six *S. rebaudiana* HMA proteins (SrHMA3, SrHMA4, SrHMA5, SrHMA6, SrHMA11, and SrHMA12) were all predicted to interact with multiple metal transporters (Figure 6). Of these, all six members were predicted to interact with the copper chaperone for superoxide dismutase (CCS). According to the STRING database, CCS is annotated as chloroplastic/cytosolic, whereas the SrHMA proteins are predominantly predicted to localize to the plasma membrane. Given this difference in subcellular localization, the predicted interactions between SrHMA proteins and CCS may reflect transient or indirect functional associations that occur during protein maturation and trafficking, rather than stable interactions at the plasma membrane. In addition, SrHMA12 and SrHMA6 had the largest numbers of interacting partners, with 16 and 13, respectively. SrHMA4 interacts with only four proteins: ATX, CCH, CCS and COPT5. Based on these predictions, we hypothesize that SrHMA3, SrHMA4, SrHMA5, SrHMA6, SrHMA11, and SrHMA12 may form interaction modules with these proteins involved in metal transport and detoxification to participate in metal ion uptake and transport. However, since these PPI predictions are based on sequence homology and known interactions in model organisms rather than experimental evidence of co-expression in specific cell types, experimental validation is thus required to confirm these interactions.

**Figure 7:**
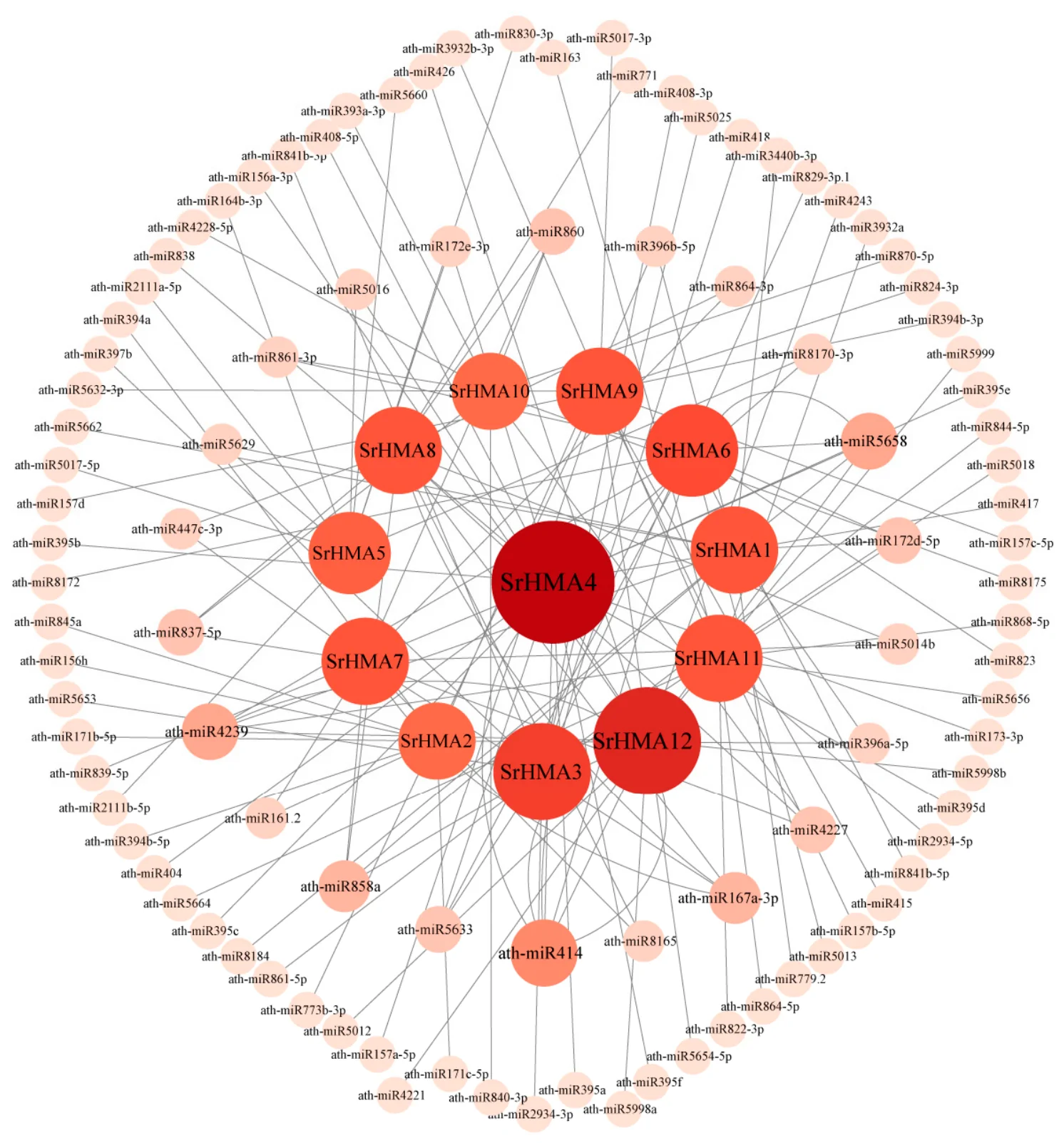
Regulation network of miRNA-*SrHMA* genes. The circles represent the indicated miRNAs or *SrHMA* genes, which are distinguished by their labels. The size and red color intensity of each circle corresponds to the number of interactions between the indicated miRNAs and *SrHMA* genes, where larger circles and darker red indicates a greater number of paired interactions of the indicated miRNAs and *SrHMA* genes. Grey lines represent interactions between miRNAs and *SrHMA* genes.

The *SrHMA* gene miRNA regulatory network predicted by psRNATarget and visualized by Cytoscape contains 99 miRNAs and 12 *SrHMA* genes, with 140 interactions (Figure 7). Most *SrHMA* genes can be targeted by multiple miRNAs. Meanwhile, some highly connected miRNAs (such as ath-miR4239, ath-miR167a-3p, ath-miR414) can regulate multiple *SrHMA* gene members. This suggests that the gene family is subject to intricate synergistic regulation at the post-transcriptional level. The analysis of network centrality revealed that miRNAs such as ath-miR4239 and ath-miR5658 were the core nodes in the network, and each interacted with four *SrHMA* genes, respectively. For instance, ath-miR5658 is predicted to simultaneously target *SrHMA1*, *SrHMA4*, *SrHMA6*, and *SrHMA12*, suggesting that this miRNA may coordinate the functions of multiple *SrHMA* members. For target genes, the *SrHMA4* gene is the most extensively regulated by miRNAs (targeted by 17 miRNAs), suggesting that its expression may be subject to tighter post-transcriptional regulation.

### 3.8. Analysis of Tissue Expression Pattern of SrHMA Genes

**Figure 8:**
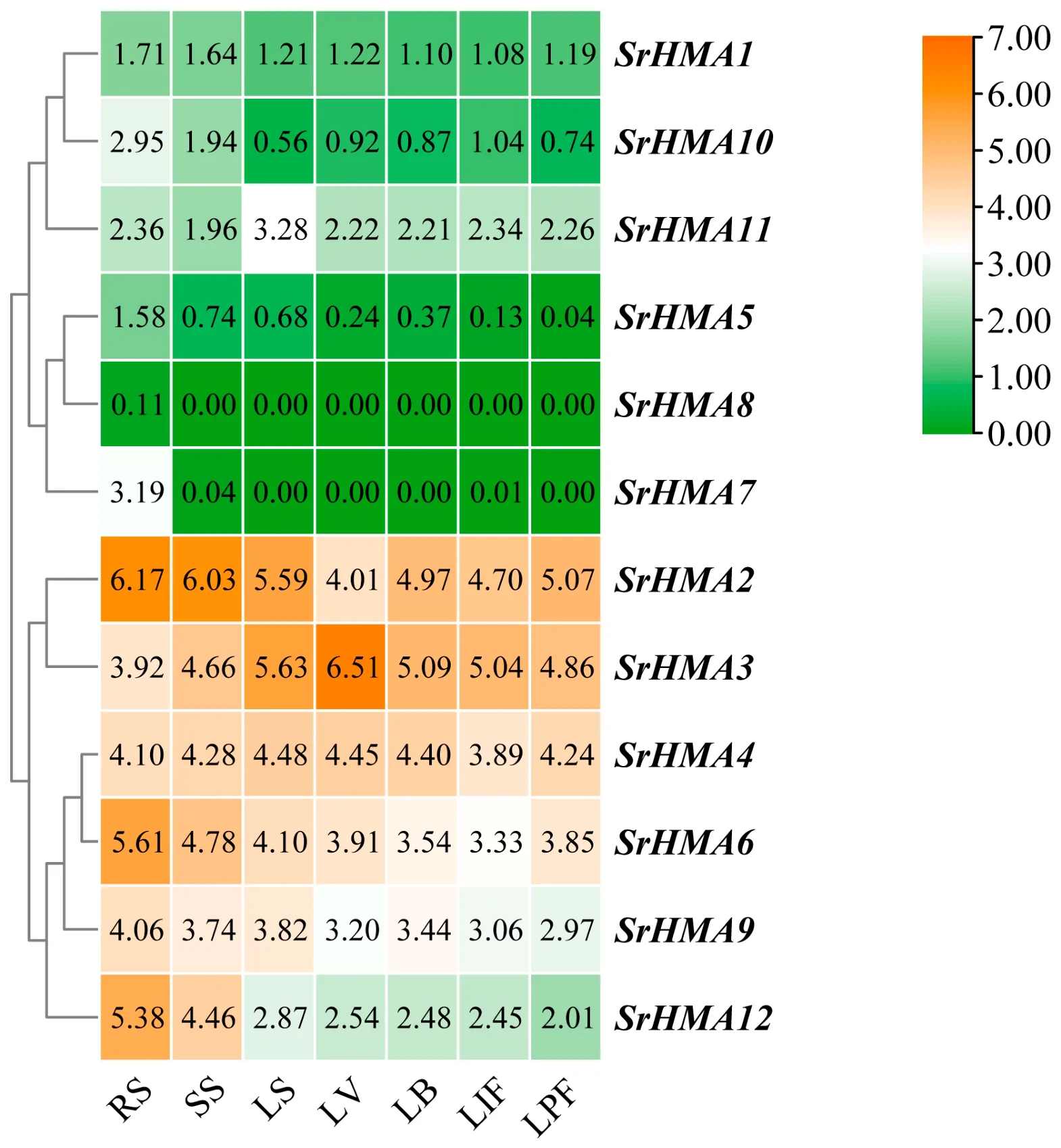
Expression profiles of *SrHMA* genes. RS: root at seedling stage; SS: stem at seedling stage; LS: leaf at seedling stage; LV: leaf at vegetative stage; LB: leaf at bud stage; LIF: leaf at initial flowering stage; LPF: leaf at peak flowering stage. The color scale at the right represents the value of log2-transformed reads per kilobase per million mapped reads. Higher expression levels are shown in orange, and lower expression levels are indicated in green.

Based on a public transcriptome dataset, we systematically analyzed the tissue-specific expression patterns of the *SrHMA* gene family in *S. rebaudiana* (Figure 8). The analysis showed that the *SrHMA* genes are mostly highly expressed in the roots, stems and leaves of the seedlings, but different genes exhibit distinct expression patterns across various tissues and developmental stages. The expression levels of *SrHMA1*, *SrHMA5*, *SrHMA8* and *SrHMA10* were extremely low in all the tested tissues, suggesting that these genes may have minor or redundant functions during the growth and development of *S. rebaudiana*. Despite the overall expression level being very low, *SrHMA1*, *SrHMA5*, *SrHMA8* and *SrHMA10* still showed the highest expression levels relative to other tissues in the RS, suggesting that they may play limited but specific roles in roots. *SrHMA2*, *SrHMA3*, *SrHMA4* and *SrHMA6* exhibited relatively high expression levels in all tissues, suggesting that they may be involved in basic metal ion transport process of *S. rebaudiana*, and they play a universal role in maintaining the homeostasis of essential trace elements and responding to metal stress. *SrHMA11* and *SrHMA4* are specifically highly expressed in the LS, while *SrHMA3* has the highest expression level in the LV. The remaining members have the highest expression level in the RS, and they may be involved in the early response and absorption regulation of heavy metals by the roots of seedlings.

### 3.9. Expression Profiling of SrHMA Genes Under Cadmium and Copper Stress

**Figure 9:**
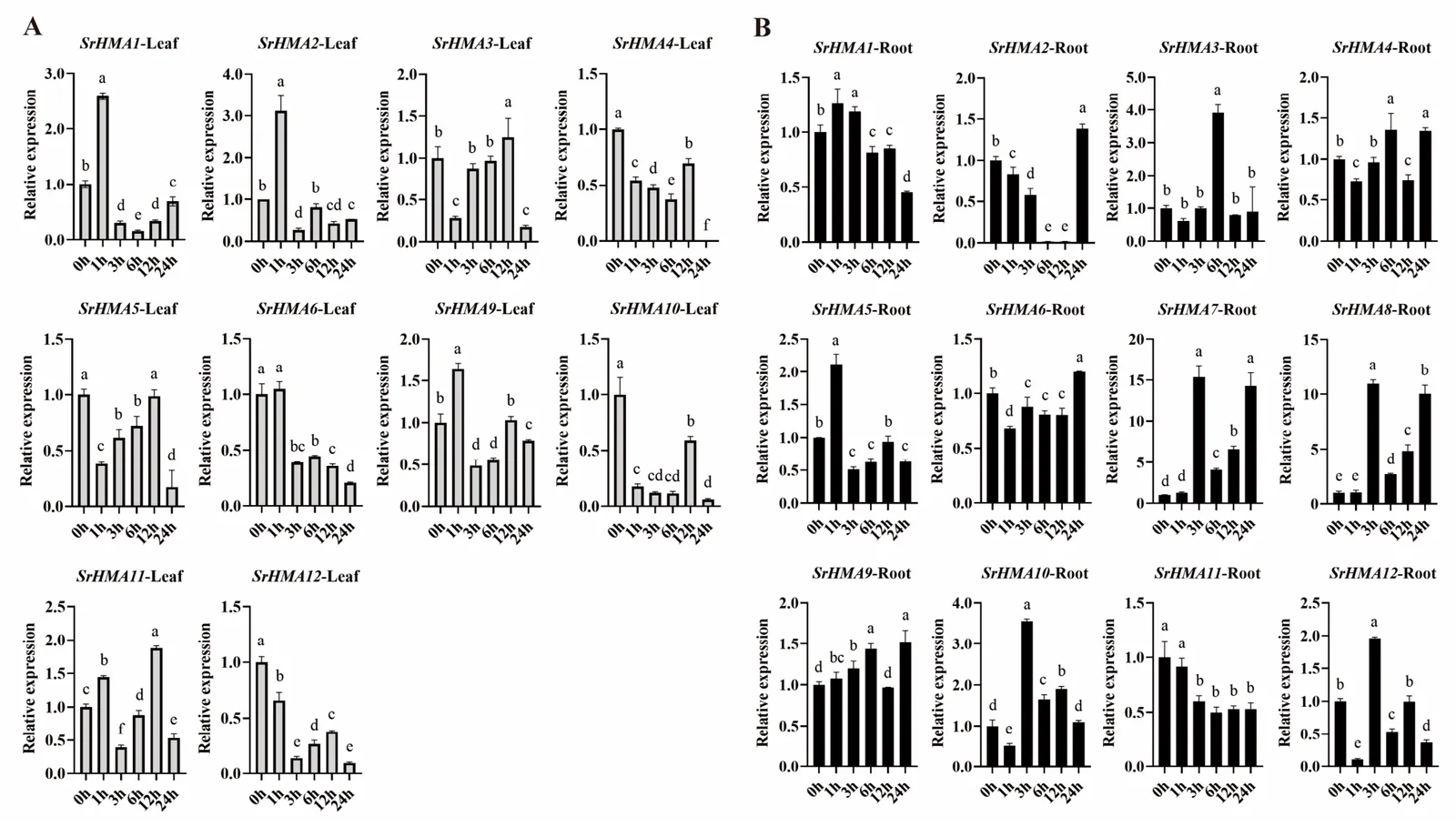
Expression analysis of the *SrHMA* genes in *S. rebaudiana* under heavy-metal cadmium treatment. (**A**) Expression of the *SrHMA* genes in the leaf. (**B**) Expression of the *SrHMA* genes in the root. The experimental data were calculated using mean ± standard error (Mean ± SE), with different lowercase letters indicating significant differences between different processing time points, which were analyzed by Duncan’s multiple range test (*p* < 0.05).

**Figure 10:**
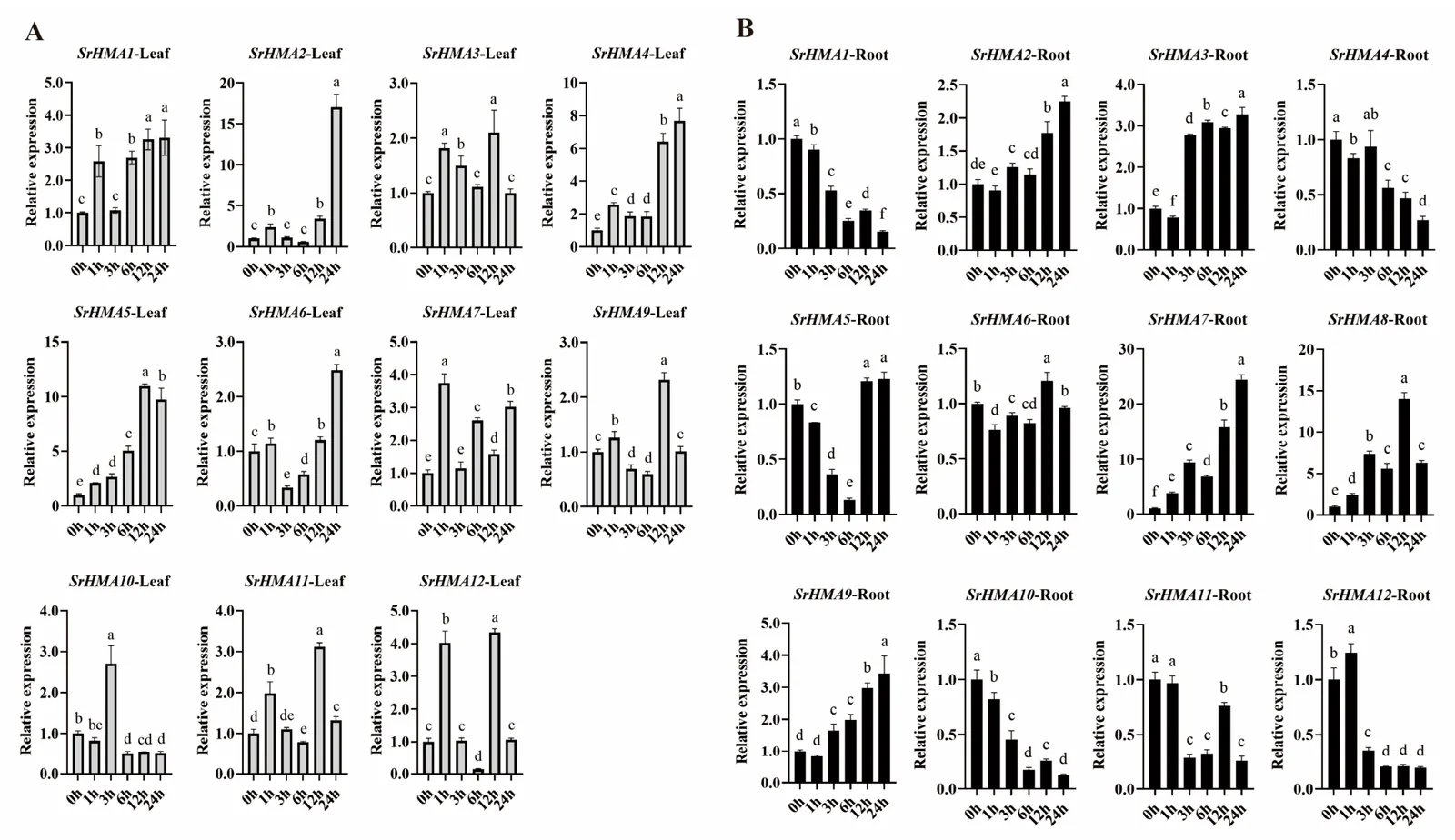
Expression analysis of the *SrHMA* genes in *S. rebaudiana* under heavy-metal copper treatment. (**A**) Expression of the *SrHMA* genes in the leaf. (**B**) Expression of the *SrHMA* genes in the root. The experimental data were calculated using mean ± standard error (Mean ± SE), with different lowercase letters indicating significant differences between different processing time points, which were analyzed by Duncan’s multiple range test (*p* < 0.05).

To investigate the transcriptional response of *SrHMA* genes to 100 μM CdCl_2_ and 721 μM CuSO_4_ treatments, we examined their expression patterns in *S. rebaudiana* using qRT-PCR. As shown in Figure 9 and Figure 10, the response of this gene family to cadmium stress exhibited significant tissue specificity and temporal dynamics (Figure 9A,B). In the leaves, most of the genes showed notable changes after 1 h of treatment. *SrHMA1*, *SrHMA2*, *SrHMA9*, and *SrHMA11* showed early and rapid induction. The expression levels of *SrHMA1* and *SrHMA2* increased to 2.7–3.0 times compared to that of the control (0 h), while that of *SrHMA9* was approximately 1.6 times and that of *SrHMA11* was approximately 1.4 times. After 3 h, these genes all decreased markedly. Notably, *SrHMA11* exhibited another peak in expression at 12 h (approximately 1.9 times higher compared to 0 h), which may be involved in the secondary regulatory response during the later stage of stress. In contrast, *SrHMA3*, *SrHMA4*, *SrHMA5*, *SrHMA10* and *SrHMA12* were inhibited within 1 h (approximately 0.17 to 0.85 times). Among them, *SrHMA3* returned to 1.0–1.4 times at 6 or 12 h but dropped sharply at 24 h. *SrHMA4* and *SrHMA5* approached the control level at 12 h but dropped to an extremely low level at 24 h. *SrHMA10* or *SrHMA12* slightly recovered at 12 h but was inhibited again. *SrHMA6* showed a continuous decrease, with its expression level being only 0.20–0.21 times compared to that of the control after 24 h of treatment. After 24 h of treatment, the expression levels of almost all *SrHMA* genes in the leaves were lower than those of the control, indicating that long-term cadmium stress exerted an overall inhibitory effect on the *SrHMA* genes in the leaves.

In roots, the *SrHMA* genes respond more strongly to cadmium stress. *SrHMA7* and *SrHMA8* reached their peaks at 3 h (approximately 15 times and 12 times, respectively), and although there was a subsequent decline, they rose again at 12 h and 24 h, showing multiple peaks. The expression levels of *SrHMA5* or *SrHMA1* showed an early upregulation at 1 h followed by a decline. The expression level of *SrHMA9* showed a slight upregulation within 1, 3, 6 and 24 h. *SrHMA3*, *SrHMA4* and *SrHMA10* showed a trend of inhibition first and then increased: a 1-h decrease, a significant recovery from 3 to 6 h, and fluctuations in the later stage. *SrHMA12* showed a sharp decline within 1 h, followed by a rapid rebound. SrHMA2 exhibited a unique pattern, with its expression level almost completely silenced from 6 to 12 h, and then it sharply increased again at 24 h, presenting a U-shaped pattern. *SrHMA11* maintained a low expression level continuously. In contrast, the expression level of *SrHMA6* showed a slight downregulation at 1 to 12 h, followed by a slight upregulation at 24 h.

The above results indicate that the expression of the *SrHMA* genes in the roots of *S. rebaudiana* changes more pronouncedly under cadmium stress compared to that in the leaves. Among them, *SrHMA3*, *SrHMA7*, *SrHMA8* and *SrHMA10* exhibited pronounced transcriptional induction in roots under cadmium stress, suggesting their potential involvement in cadmium detoxification. *SrHMA1* and *SrHMA2* showed early upregulation in leaves, while the U-shaped expression pattern of *SrHMA2* in roots implies a possible role in the later stage of stress response. However, functional validation through approaches such as heterologous expression or knockout/knockdown lines would be required to confirm the precise roles of these genes in cadmium detoxification and tolerance.

Under copper stress, the *SrHMA* genes of *S. rebaudiana* exhibited rapid and intense responses in both leaves and roots, but the dynamic patterns in the two organs were markedly different (Figure 10A,B). In the leaves, the expression levels of *SrHMA1*, *SrHMA3*, *SrHMA4*, and *SrHMA7* generally showed an upward trend, with significant upregulation (1.5 to 4 times) observed 1 h after copper stress treatment, reaching a peak level at 1, 12, and 24 h, respectively. The expression levels of *SrHMA2* and *SrHMA6* exhibited a dynamic trend of initial upregulation, followed by downregulation and a subsequent secondary upregulation, with peak expression observed at 24 h (2 and 15 times, respectively). The expression level of *SrHMA5* continuously increased over the treatment time, rising from 2 times at 1 h to 11 times at 12 h, and remaining at a relatively high level of approximately 10 times at 24 h. The expression level of *SrHMA9*, *SrHMA11*, and *SrHMA12* showed an overall trend of increasing, then decreasing, followed by another increase and subsequent decrease, reaching its peak at 12 h (2, 3, or 4 times, respectively). In contrast, the expression level of *SrHMA10* was significantly upregulated at 3 h (2 times), while the expression levels at other time points were lower than those in the control (0 h). These dynamic expression patterns illustrate a classical transition from an early stress response at 1 h to adaptive recovery in the later phase, which may be associated with transcriptional reprogramming and the reestablishment of cellular homeostasis.

The response of the *SrHMA* genes in the roots to copper stress is more intense than that in the leaves. *SrHMA7* and *SrHMA8* were induced to approximately 4 times and 2.5 times, respectively, within 1 h compared to that of the control (0 h), and then continued to rise. By 12 or 24 h, their expression level reached peak values of 25 times and 14 times, respectively. The expression levels of *SrHMA2*, *SrHMA3*, *SrHMA5*, and *SrHMA9* showed a pattern of initial suppression, followed by an increase. Among them, the expression level of *SrHMA2* gradually increased from 3 h and reached 2.2 times at 24 h; the expression level of *SrHMA3* was suppressed at 1 h and maintained a high expression level (3 to 3.4 times) from 3 h onwards; the expression level of *SrHMA5* was lowest at 6 h, followed by a slight increase at 12 and 24 h compared to the control (0 h); the expression level of *SrHMA9* slowly increased from 3 to 6 h and remained at around 3 times from 12 to 24 h. The expression level of *SrHMA1*, *SrHMA4*, *SrHMA10*, *SrHMA11*, and *SrHMA12* showed an overall decreasing trend, reaching the lowest level at 24 h. In contrast, the expression level of *SrHMA6* showed minor fluctuations.

In summary, under copper stress, the *SrHMA* gene family in *S. rebaudiana* exhibited a rapid response, organ specificity, and functional differentiation. The response amplitude of the *SrHMA* genes in the root was much greater than that in the leaves, and more genes showed late recovery or continuous high expression. *SrHMA5* is continuously upregulated in leaves, possibly participating in the continuous detoxification of copper; *SrHMA2* shows extremely high levels in both leaves and roots at a later stage, suggesting its crucial role in response to copper stress; *SrHMA7* and *SrHMA8* are strongly induced in the roots, and may be the core members of copper detoxification in the roots. These results provide important evidence for the subsequent screening of key candidate genes involved in the response of *S. rebaudiana* to copper stress.

Notably, the observed temporal differences in gene activation may partly result from expression in distinct cell types within the same organ (e.g., epidermis or vasculature). For instance, early-responding genes may be preferentially expressed in epidermal cells, whereas late-responding genes may be active in vascular tissues. Since our qRT-PCR data were obtained from whole roots and leaves, they cannot resolve such cell-type specificity. Future studies using cell-type-specific approaches, such as in situ hybridization or single-cell RNA-seq, would be required to clarify the spatial and temporal coordination of *SrHMA* gene expression under cadmium or copper stress.

## 4. Discussion

The *SrHMA* gene family plays a key role in heavy-metal homeostasis in plants by transporting heavy metals through ATP hydrolysis, functioning as an important class of heavy-metal transporters [26,42,43]. To date, this gene family has been studied in multiple species, including the model plant *A. thaliana* [44], crop plants such as *G. max* [26] and *Arachis hypogaea* [45], as well as horticultural species like *Populus trichocarpa* [24], *Medicago sativa* [46], and *H. macrophylla* [25]. However, the *HMA* genes in *S. rebaudiana* had not been previously identified. This study reports the first identification of twelve *HMA* genes in *S. rebaudiana*. This number is slightly higher than those in *A. thaliana*, *O. sativa*, and *H. macrophylla*, similar to *Areca catechu* [47] and *Linum usitatissimum* [48], but considerably lower than in polyploid species such as *G. max* [26] and *Brassica napus* [49]. Diploid plants vary significantly in genome size, but their number of *HMA* genes does not increase markedly with genome expansion, suggesting no strong positive correlation between *HMA* gene count and genome size. In contrast, polyploid species generally possess more *HMA* genes, indicating that polyploidization may play a more important role in the expansion of this gene family. Phylogenetic analysis showed that the *SrHMA* genes, like those in *A. thaliana* and *O. sativa*, can be divided into two distinct subclades, implying that the divergence of the *HMA* family predated the major speciation events in plants. Furthermore, members within the same clade may potentially perform similar functions. For example, *AtHMA5,* which is predominantly expressed in roots, induced specifically by copper, and essential for copper detoxification [17], clusters together with *SrHMA7*, *SrHMA8*, and *SrHMA10*. These *SrHMA* genes are also primarily root-expressed and are hypothesized to play similar roles in copper detoxification in *S. rebaudiana*, although this prediction requires further experimental validation.

All *SrHMA* genes contain eight identical conserved motifs, although each subgroup also possesses specific fixed motifs. Certain genes exhibit unique motif compositions, with the Cu/Ag subgroup showing considerable variation in the number of conserved motifs, indicating both functional conservation and diversity within the *SrHMA* gene family. Each SrHMA protein contains an E1-E2_ATPase domain, a hydrolase domain, and at least one HMA domain at the N-terminus, similar to the domain architecture observed in *Arabidopsis*. Exons are important regulatory elements in eukaryotic gene expression. Their gain or loss is common in evolution and may lead to proteins with altered domain organization and sequence features, potentially contributing to functional diversification [50,51]. Among the Zn/Co/Cd/Pb subgroup, all members contain nine exons except *SrHMA1*, which has 11. In contrast, the number of exons in the Cu/Ag subgroup varies widely from 6 to 17, with *SrHMA3* and *SrHMA4* containing the most (14 and 17 exons, respectively), suggesting they may perform specialized functions. The initiation and expression level of gene transcription are dynamically regulated by cis-acting elements in the promoter region, and promoter analysis can reflect a gene’s responsiveness to various biotic and abiotic stimuli [52,53]. As observed in *HMA* genes of most plant species [41,54], the promoters of *SrHMA* family members contain abundant cis-elements related to abiotic stress and light responses. With the exception of *SrHMA11*, which lacks growth-related regulatory elements, all other members possess such motifs, suggesting that these genes may be involved not only in stress adaptation but also in growth and developmental processes.

Gene segmental duplication and tandem duplication are key mechanisms driving the expansion of gene families, playing an important role in organismal adaptation and genome evolution [55,56]. During the evolution of *S. rebaudiana*, the species underwent several polyploidization events shared by all core eudicots (WGT-γ), as well as lineage-specific whole-genome triplication (WGT-1) and duplication (WGD-2) events characteristic of Asteraceae [57]. Intra-species synteny analysis identified two segmentally duplicated gene pairs within the twelve *SrHMA* members. The Ka/Ks ratios for these two duplicated pairs were less than 1, indicating that the *SrHMA* genes have undergone purifying selection during evolution. Inter-species synteny analysis revealed that *SrHMA* genes shared syntenic relationships exclusively with dicots, but not with monocots. These results suggest that the *HMA* gene family likely experienced genomic duplication events specifically in dicot lineages, leading to structural reorganization and functional diversification, whereas the family remained relatively conserved in monocots.

Subcellular localization is a key indicator of protein function. In *Arabidopsis*, AtHMA1, AtHMA6, and AtHMA8 are localized to chloroplasts and are involved in chloroplast copper homeostasis [42]. Predictive analysis of SrHMA protein sequences revealed that only SrHMA3, a homolog of AtHMA8, is predicted to localize to the chloroplast. Most other members of the SrHMA family are predicted to reside in the plasma membrane, while SrHMA9 is forecast to localize to the vacuole membrane, consistent with subcellular localization patterns observed for HMA proteins in other species [25,54]. From a phylogenetic perspective, all members of the Zn/Co/Cd/Pb subgroup are predicted to localize to the plasma membrane, whereas predictions within the Cu/Ag subgroup show greater variation. These predictions imply that even within the same subfamily, homologous proteins may exhibit divergent subcellular localization, potentially reflecting functional diversification. However, definitive subcellular localization requires experimental validation (e.g., via transient expression of GFP-fusion proteins). Gene expression profiles can provide insights into the potential functions of *SrHMA* genes. Based on available RNA-seq data, we analyzed the expression patterns of *SrHMAs* in roots, stems, and leaves at the seedling stage, as well as in leaves at different developmental stages. The results indicate that most *SrHMA* genes show organ-specific expression, with the highest levels generally observed in roots—a pattern consistent with *HMA* genes in wheat and alfalfa [41,46], suggesting a potential role for *SrHMA* genes as heavy-metal transporters in root tissues.

Analysis of the expression patterns of the *SrHMA* genes in leaves and roots under cadmium and copper stress revealed that this gene family can rapidly respond to both heavy-metal stresses, respectively, but with marked differences in response amplitude, dynamic trends, and organ specificity. In the leaves, under cadmium stress, most genes showed early induction followed by rapid decline, and were generally inhibited after 24 h. Among them, *SrHMA1* and *SrHMA2* showed the most pronounced upregulation under the tested conditions. Under copper stress, the induction amplitude of *SrHMA* genes in leaves was greater, and they presented various patterns such as continuous increase, double peaks, or initial suppression followed by increase. This suggests that copper stress may activate *HMA* genes in leaves more persistently and intensely, but this hypothesis next requires further experimental testing. At the root, both stresses induced extremely strong responses. Under cadmium stress, *SrHMA7* and *SrHMA8* in the roots showed extremely high expression levels within 3 h and showed multiple peaks; *SrHMA2* exhibited a unique “U-shaped” pattern. Under copper stress, both *SrHMA7* and *SrHMA8* were strongly induced. However, under copper treatment, *SrHMA7* reached its peak at 24 h, while *SrHMA8* reached its peak at 12 h and then declined. Furthermore, copper stress strongly induced the “V-shaped” recovery of *SrHMA5* and the subsequent sustained high expression of *SrHMA3* and *SrHMA9*. Notably, *SrHMA2* exhibited extremely high levels in both stress conditions, suggesting a potential crucial role in the later stage of heavy-metal stress that needs to be confirmed. Interestingly, *SrHMA7* and *SrHMA8* were strongly induced in the roots under cadmium and copper stress, showing multiple peaks or continuous increases. However, no reports have been found in model plants such as *Arabidopsis* and *O. sativa* regarding the homologous genes of *SrHMA7/8* having such a strong response to heavy-metal stress [17]. Given this unique and robust expression pattern, these two genes deserve particular attention in future functional studies, but this needs to be validated through functional assays such as heterologous expression or knockout lines. While *SrHMA5* is continuously upregulated in leaves in response to copper stress, it shows a “V-shaped” recovery in roots in response to copper stress. Its response to cadmium stress is relatively weak, suggesting a potential metal-specific role that awaits further validation. Overall, these bioinformatics predictions provide a basis for generating testable hypotheses; however, all predicted functions and regulatory roles of *SrHMA* genes require experimental validation through approaches such as heterologous expression, mutant complementation, or gene knockout/knockdown lines.

## 5. Conclusions

In summary, this study presents a comprehensive bioinformatic analysis of the *HMA* gene family in *S. rebaudiana*, leading to the identification of twelve *SrHMA* genes, which were phylogenetically classified into two subgroups: Zn/Co/Cd/Pb and Cu/Ag. Members within the same subgroup exhibited conserved motif patterns, while variations were observed between subgroups. Synteny analysis suggested that the expansion of the *SrHMA* family was primarily driven by segmental duplication events, suggesting evolutionary signs of potential functional divergence. Promoter analysis revealed the presence of abundant cis-regulatory elements associated with abiotic stress, hormone response, and light responsiveness in all *SrHMA* genes. Analysis of organ expression patterns and stress responses revealed that *SrHMA* genes exhibit organ-specific expression, with marked differences in response amplitude, dynamic trends, and organ distribution under cadmium and copper stress. These findings provide a foundation for subsequent studies on the functions of the *SrHMA* gene and the molecular mechanism of cadmium and copper adaptation in *S. rebaudiana*. In particular, the strong and specific induction of *SrHMA7* and *SrHMA8* under heavy-metal stress makes them promising candidates for further functional characterization via transgenic approaches, such as heterologous expression in yeast or *Arabidopsis*, or the generation of knockout/overexpression lines in *S. rebaudiana*, which would help elucidate their precise roles in metal detoxification and tolerance.

## Figures and Tables

**Table 1:** The analysis of physicochemical properties and subcellular localization of *S. rebaudiana SrHMA* gene family members.

Gene Name	Gene ID	Amino Acid Length (aa)	MWs (kDa)	pI	II	GRAVY	Subcellular Localization
*SrHMA1*	Streb.1G033920.1	807	86,971.8	6.64	29.87	0.179	plasma membrane
*SrHMA2*	Streb.1G034300.1	1011	109,163.2	5.23	32.33	0.238	plasma membrane
*SrHMA3*	Streb.2G030460.1	763	82,105.74	6.12	42.19	0.14	chloroplast
*SrHMA4*	Streb.2G032880.1	938	98,706.41	7.53	37.02	0.194	plasma membrane
*SrHMA5*	Streb.2G044220.1	862	93,304.65	5.99	33.21	0.37	plasma membrane
*SrHMA6*	Streb.3G014270.1	956	103,964.3	6.68	28.28	−0.081	plasma membrane
*SrHMA7*	Streb.3G016680.1	987	107,054.09	6.3	31.18	0.189	plasma membrane
*SrHMA8*	Streb.3G016730.1	929	100,905.05	6.76	32.74	0.159	plasma membrane
*SrHMA9*	Streb.3G016780.1	960	103,596.95	5.36	34.39	0.217	vacuole membrane
*SrHMA10*	Streb.10G020560.1	990	107,843.05	5.99	35.41	0.168	plasma membrane
*SrHMA11*	Streb.10G024800.1	967	104,085.8	5.84	33.66	0.267	plasma membrane
*SrHMA12*	Streb.11G026310.1	1149	127,330.27	6.27	33.27	−0.276	plasma membrane

## Data Availability

The original contributions presented in this study are included in the article/Supplementary Material. Further inquiries can be directed to the corresponding authors.

## Supplementary Materials

The following supporting information can be downloaded at: https://www.mdpi.com/article/10.3390/cells15181646/s1, Figure S1. Multi-sequence alignment of HMA protein in *S. rebaudiana*, *A. thaliana*, and *O. sativa*. Figure S2. Secondary structure analysis of the SrHMA protein in S. rebaudiana. Table S1. Primers used in this study. Table S2. The registration numbers of HMA proteins in different species. Table S3. The secondary structure of SrHMA proteins. Table S4. The Ka/Ks ratios analysis of paralog gene pair of *SrHMA* gene family.
