# Supplementary material for: Genome-Wide Identification and Expression Analysis of the Heavy-Metal ATPase (HMA) Gene Family Reveal Their Correlation with Copper and Cadmium Stress Adaptation in Stevia rebaudiana
Source: Cells. 2026 Sep 11;15(18):1646. doi: 10.3390/cells15181646 (PMC13605984; doi:10.3390/cells15181646)
Supplement: Supplementary file 1 [file cells-15-01646-s001.zip › cells-4496970-supplementary.pdf]

Supplementary Materials

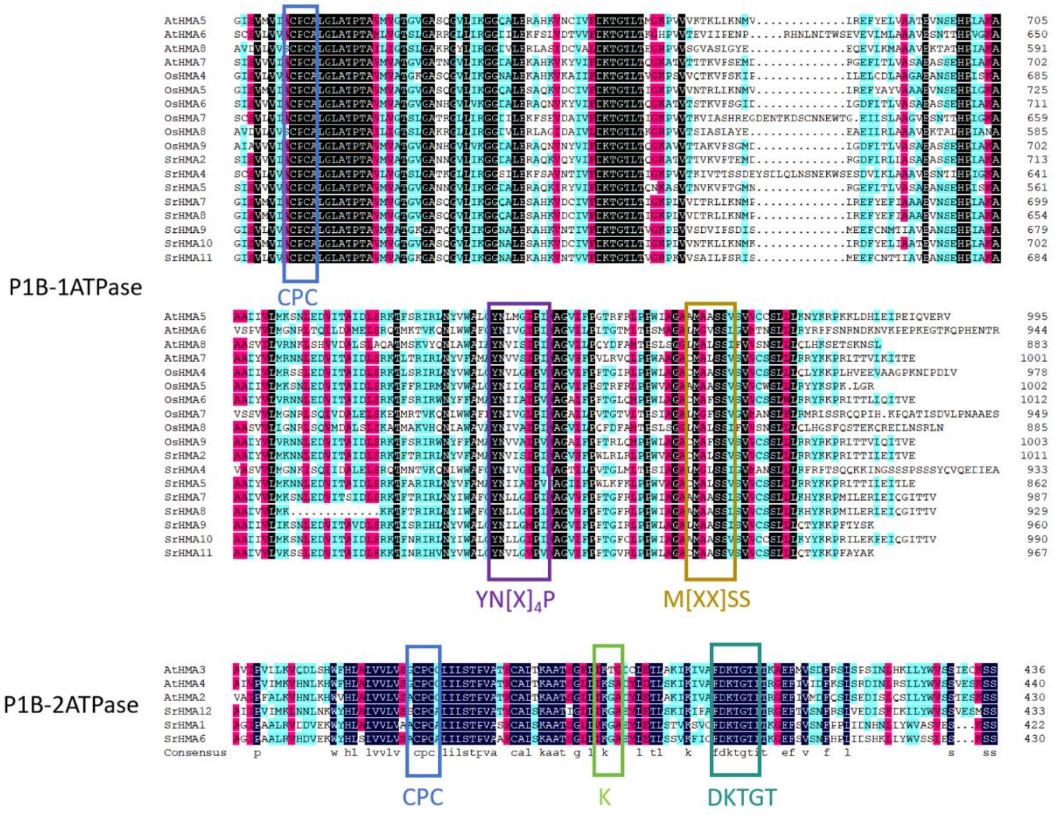

Supplemental Figure S1. Multi-sequence alignment of HMA protein in *S. rebaudiana*, *A. thaliana*, and *O. sativa*.

Black, pink and gray backgrounds respectively indicate 100%, ⩾80% and ⩾60% amino acid homology. The blue box, purple box, brown box, cyan box and green box respectively represent the CPC, YN[X] 4P, M[XX]SS, K and DKTGT feature motifs.

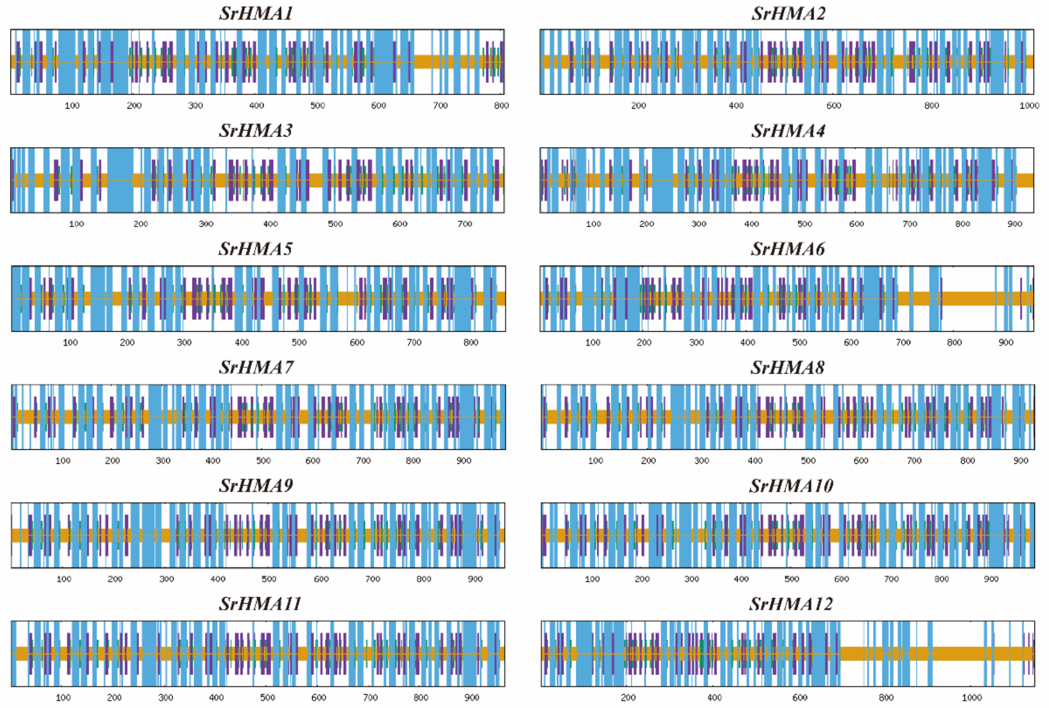

Supplemental Figure S2. Secondary structure analysis of the SrHMA protein in *S. rebaudiana*.

Secondary structure analysis of the SrHMA1 to SrHMA12 protein. Blue: α-helix; purple: extended strand; yellow: random coil; green: β-turn.

Supplemental Table S1. Primers used in this study.

Name	Primer sequence (5'-3')
Actin-F	GTATTGCCGACAGGATGAGTAAGG
Actin-R	TCTGTTGGAAGGTGCTGAGTGA
SrHMA1-F	TGGGTTGCAAGCGTAGAGAG
SrHMA1-R	GCGAGTGTAGACCCGACAAA
SrHMA2-F	CGGTGATGATCTCGAGGACG
SrHMA2-R	ATGCTGCAACAAGGCAACAG
SrHMA3-F	CAGGCGAGTCACTTCCTGTT
SrHMA3-R	AGGTGCTTCACGTCCTTGAG
SrHMA4-F	AGACCGTGTTCCTGCTGATG
SrHMA4-R	CAGGAGCTTCCCTGCTTTGA
SrHMA5-F	ATTGCAATAGAGGCTGCCGA
SrHMA5-R	GAATGCCAGCCGATACAGGA
SrHMA6-F	TTGCAAACGAAACCGGACAC
SrHMA6-R	AGAACCGCAGCCCAAACTAA
SrHMA7-F	AAGCCCAAGTGGCTTTAGCAAC
SrHMA7-R	CGGGAAGTGCGTCAAGAGATT
SrHMA8-F	CGAGTCCGCTTTAACTGGTTTACAA
SrHMA8-R	CGGTTAGATCCGCTTTGTAAGAACG
SrHMA9-F	CTTTGAGGAGCACCCTGGAG
SrHMA9-R	TCAACCGCCACGATTACACA
SrHMA10-F	GTGTTACGAGCTGCGGTTTC
SrHMA10-R	GTATCGCGGTATCGGGTGTT
SrHMA11-F	ATTGAAGCGGCGGATGTAGT
SrHMA11-R	CTCCGGTGAACGGAAACAGA
SrHMA12-F	CGGCTTTCTTGATACAAAGAAAGAGATGA
SrHMA12-R	CTATTTGAAGCGGGGAAACAAGAACG

Supplemental Table S2. The registration numbers of HMA proteins in different species.

Species	Protein name	Accession number/Locus ID
Arabidopsis thaliana	AtHMA1	AT4G37270.1
	AtHMA2	AT4G30110.1
	AtHMA3	AT4G30120.1
	AtHMA4	AT2G19110.1
	AtHMA5	AT1G63440.1
	AtHMA6	AT4G33520.2
	AtHMA7	AT5G44790.1
	AtHMA8	AT5G21930.1
Oryza sativa	OsHMA1	LOC_Os06g47550.1
	OsHMA2	LOC_Os06g48720.1
	OsHMA3	LOC_Os07g12900.1
	OsHMA4	LOC_Os02g10290.1
	OsHMA5	LOC_Os04g46940.1
	OsHMA6	LOC_Os02g07630.1
	OsHMA7	LOC_Os08g37950.1
	OsHMA8	LOC_Os03g08070.1
	OsHMA9	LOC_Os06g45500.1

Supplemental Table S3. The secondary structure of SrHMA proteins.

Gene name	α-helix (%)	β-turn (%)	Random coil (%)	Extended strand (%)
SrHMA1	45.35	7.06	30.73	16.85
SrHMA2	42.83	6.63	33.63	16.91
SrHMA3	41.94	6.95	34.08	17.04
SrHMA4	43.39	6.4	33.37	16.84
SrHMA5	43.62	7.08	32.25	17.05
SrHMA6	40.38	5.96	39.75	13.91
SrHMA7	43.36	6.99	33.23	16.41
SrHMA8	41.66	7.21	33.80	17.33
SrHMA9	42.60	7.29	33.23	16.88
SrHMA10	45.25	6.97	30.81	16.97
SrHMA11	43.23	7.14	32.57	17.06
SrHMA12	36.64	4.70	47.00	11.66

Supplemental Table S4. The Ka/Ks ratios analysis of paralog gene pair of *SrHMA* gene family.

Seq1	Seq2	Ka	Ks	Ka/Ks	Effective length	AverageS-sites
SrHMA11	SrHMA7	0.4061091750359287	3.721523369350475	0.10912444575265633	2853	682.6666666666665
SrHMA4	Streb.6G009190.1	0.31403071157880025	0.8230695366814524	0.3815360641883866	549	140.41666666666666
